# ^18^F-AV1451 PET imaging and multimodal MRI changes in progressive supranuclear palsy

**DOI:** 10.1007/s00415-019-09566-9

**Published:** 2019-10-22

**Authors:** Nicolas Nicastro, Patricia Vazquez Rodriguez, Maura Malpetti, William Richard Bevan-Jones, P. Simon Jones, Luca Passamonti, Franklin I. Aigbirhio, John T. O’Brien, James B. Rowe

**Affiliations:** 1 Department of Psychiatry, University of Cambridge, Cambridge, UK; 2 Department of Clinical Neurosciences, Geneva University Hospitals, Geneva, Switzerland; 3 Department of Clinical Neurosciences, University of Cambridge, Herchel Smith Building, Forvie Site, Robinson Way, Cambridge Biomedical Campus, Cambridge, CB2 0SZ UK; 4 Consiglio Nazionale Delle Ricerche (CNR), Istituto Di Bioimmagini E Fisiologia Molecolare (IBFM), Milano, Italy; 5 Wolfson Brain Imaging Center, University of Cambridge, Cambridge, UK; 6 Medical Research Council Cognition and Brain Sciences Unit, Cambridge, UK

**Keywords:** Tau, PET, MRI, Diffusion tensor imaging, Progressive supranuclear palsy

## Abstract

**Objectives:**

Progressive supranuclear palsy (PSP) is characterized by deposition of straight filament tau aggregates in the grey matter (GM) of deep nuclei and cerebellum. We examined the relationship between tau pathology (assessed via ^18^F-AV1451 PET) and multimodal MRI imaging using GM volume, cortical thickness (CTh), and diffusion tensor imaging (DTI).

**Methods:**

Twenty-three people with clinically probable PSP-Richardson’s syndrome (age 68.8 ± 5.8 years, 39% female) and 23 controls underwent structural 3 T brain MRI including DTI. Twenty-one patients also had ^18^F-AV1451 PET imaging. Voxelwise volume-based morphometry, surface-based morphometry, and DTI correlations were performed with ^18^F-AV1451 binding in typical PSP regions of interest (putamen, thalamus and dentate cerebellum). Clinical impairment was also assessed in relation to the different imaging modalities.

**Results:**

PSP subjects showed GM volume loss in frontotemporal regions, basal ganglia, midbrain, and cerebellum (FDR-corrected *p* < 0.05), reduced CTh in the left entorhinal and fusiform gyrus (*p* < 0.001) as well as DTI changes in the corpus callosum, internal capsule, and superior longitudinal fasciculus (FWE-corrected *p* < 0.05). In PSP, higher ^18^F-AV1451 binding correlated with GM volume loss in frontal regions, DTI changes in motor tracts, and cortical thinning in parietooccipital areas. Cognitive impairment was related to decreased GM volume in frontotemporal regions, thalamus and pallidum, as well as DTI alteration in corpus callosum and cingulum.

**Conclusion:**

This cross-sectional study demonstrates an association between in vivo proxy measures of tau pathology and grey and white matter degeneration in PSP. This adds to the present literature about the complex interplay between structural changes and protein deposition.

## Introduction

Progressive supranuclear palsy (PSP) encompasses a broad spectrum of motor, cognitive, and behavioural impairments. These include akinesia, postural instability with early falls, oculomotor deficits, fronto-executive dysfunction, and neuropsychiatric features such as apathy and impulsivity [1, 2]. Neuropathological studies have shown that PSP is characterized by intra-neuronal and astrocytic aggregation of toxic microtubule-associated protein tau (of four-repeat tau isoforms, with straight filaments) [3, 4]. Using ^18^F-AV1451 positron emission tomography (PET) allows to quantify and localise in vivo tau burden. ^18^F-AV1451 binding is consistently increased in the basal ganglia, thalamus, and dentate cerebellum in patients with PSP relative to controls [5–7], mirroring its neuropathological distribution [4]. Albeit ^18^F-AV1451 affinity for tau inclusions in non-AD tauopathies is less than in AD [8–10] and is not specific to tau [11], the distribution of ^18^F-AV1451 shows distinctive features in PSP [6]. Therefore, where high clinic-pathological correlations indicate likely PSP tau pathology, ^18^F-AV1451 can be used to quantify it and compare to other disease processes such as white matter pathology.

Multimodal structural imaging in PSP usually shows grey matter (GM) volume loss in midbrain, striatum and frontal cortex, cortical thickness (CTh) reduction in frontal areas, and white matter damage in cerebellar peduncles, thalamic radiations and corpus callosum [12–18].

Despite the separate insights from ^18^F-AV1451 PET and structural imaging, the relationship between tau burden and grey/white matter degeneration in PSP has remained unknown. The aim of the study was, therefore, to assess GM volume, CTh, and diffusion tensor imaging (DTI) changes in PSP compared to Controls; second, to perform correlation analyses of structural MRI/DTI changes with ^18^F-AV1451 in high-binding regions previously shown to be involved in PSP [6]. In addition, we assessed motor and cognitive clinical correlations of both ^18^F-AV1451 binding and structural MRI/DTI imaging. Considering the atrophy and extensive white matter damage observed in PSP in the previous studies, we predicted a GM volume loss and microstructural disruption related to higher ^18^F-AV1451 binding and that the severity of these changes is related to clinical impairment.

## Methods

### Participants

The present study is part of the Neuroimaging of Inflammation in MemoRy and Other Disorders (NIMROD) protocol [19]. We included 23 participants with probable PSP (PSP-Richardson syndrome) who were recruited according to NINDS-SPSP 1996 criteria [3] but reconfirmed as meeting the current clinical diagnostic criteria of probable PSP-RS [2]. 23 similarly aged healthy controls were also recruited, with MMSE > 26/30, absence of regular memory complaints, and no history of major neurological, psychiatric, or significant medical illness. Patients were identified from the regional specialist PSP-clinic at the Cambridge University Hospitals NHS Trust. Healthy controls were recruited via the Dementias and Neurodegenerative Diseases Research Network volunteer register. Informed written consent was obtained in accordance with the Declaration of Helsinki. The study received a favourable opinion from the East of England Ethics Committee (Cambridge Central Research, Ref. 13/EE/0104). Clinical and cognitive assessment included mini-mental state examination (MMSE), revised Addenbrooke’s Cognitive Examination (ACER), and the PSP rating scale (PSP-RS) for PSP subjects [20].

### MRI acquisition and preprocessing

Participants underwent MRI imaging acquired on a 3 T scanner (Siemens Magnetom Tim Trio) using a magnetization-prepared rapid gradient echo (MPRAGE) T1-weighted sequence (repetition time = 2300 ms, echo time = 2.98 ms, field of view = 240 × 256 mm^2^, 176 slices, flip angle = 9°, isotropic 1 mm voxels).

GM volume and CTh were assessed using Computational Anatomy Toolbox 12 (CAT12, Structural Brain Imaging Group, University of Jena, Germany) running in Matlab R2018b version 9.5 (MathWorks Inc., Sherborn, MA, USA). CAT12 allows volume-based morphometry (VBM) evaluation by providing the voxelwise estimation of the local amount or volume of a specific tissue compartment [21]. The automated pipeline incorporates different preprocessing steps, including spatial registration to a reference brain, tissue segmentation into GM, white matter and CSF, and bias correction of intensity non-uniformities. Smoothing was performed using a full-width at half maximum (FWHM) of 8 mm as recommended. CAT12 can additionally assess CTh and central surface of the left and right hemispheres based on the projection-based thickness method [22]. Using a tissue segmentation to estimate the white matter distance, it then projects the local maxima (which is equal to the CTh) to other GM voxels by using a neighbour relationship described by the white matter distance. Projection-based thickness allows the handling of partial volume information, sulcal blurring, and sulcal asymmetries without explicit sulcus reconstruction [22]. Topological correction, spherical mapping, and spherical registration are performed to obtain vertexwise CTh. Finally, surface maps are smoothed using a 15 mm-FWMH for group comparisons and clinical/imaging correlations.

Diffusion-weighted images were acquired with a 65-direction encoding scheme, 2 mm thickness, TE = 106 ms, TR = 11,700 ms, field of view = 192 × 192 mm^2^. 64 volumes were acquired with a *b* value of 1000 s/mm^2^ following an initial volume with a *b* value of 0 s/mm^2^. The data were preprocessed with the FSL 6.0 software package (https://www.fmrib.ox.ac.uk/fsl) using FSL Diffusion Toolbox. First, the series was adjusted for head movement and eddy currents using eddy and realigned to the b0 image. A brain mask was then produced by applying the Brain Extraction Tool (*BET*) to the (mean) b0 image. *DTIfit* was next used to independently fit the diffusion tensor at each voxel, resulting in the derivation of fractional anisotropy (FA), mean (MD) and radial diffusivity (RD) maps. Tract-based spatial statistics (TBSS) was then used to align each subject’s FA image to a pre-identified target FA image (FMRIB_58) [23], followed by affine registration into the Montreal Neurological Institute (MNI) MNI152 space. A mean FA image and skeleton were created from all subjects and each individual's FA image was then projected onto the skeleton, with a threshold of 0.2 applied to the mean skeleton to include white matter tracts that were common across all subjects and to exclude voxels that may contain GM or CSF. The aligned DTI parameter map of each subject was then back-projected onto the mean skeleton. In addition, other DTI parameters (i.e., MD and RD) were aligned by applying the original FA non-linear spatial transformations to the corresponding datasets and projecting them onto the mean FA skeleton.

### ^18^F-AV1451 PET acquisition and preprocessing

21 PSP participants underwent ^18^F-AV-1451 PET imaging. The radioligand was prepared at the Wolfson Brain Imaging Centre, University of Cambridge, UK. PET scanning was performed on a GE Advance or a GE Discovery 690 PET/CT (General Electric Healthcare, Chicago, Illinois, USA). A 15-min ^68^Ge/^68^ Ga transmission scan was used for attenuation correction. The emission protocol was as follows: 90 min dynamic imaging following a 370 MBq ^18^F-AV1451 injection. Each emission image series was aligned using SPM8 to correct for patient motion during data acquisition (www.fil.ion.ucl.ac.uk/spm/software/spm8). The mean aligned PET images were rigidly registered to the T1-weighted image to extract values from both the Hammers atlas regions of interest (ROIs) and those in a reference tissue defined in the superior cerebellar GM using a 90% GM threshold on the GM probability map produced by SPM8 smoothed to PET resolution [24]. Regional PET data were corrected for CSF partial volumes. ^18^F-AV1451 non-displaceable binding potential (BP_ND_) was determined for each ROI using a basis function implementation of the simplified reference tissue model [25]. As shown in a previous publication [6], our PSP cohort had significantly higher ^18^F-AV1451 BP_ND_ in the putamen, pallidum, thalamus, midbrain, and cerebellar dentate gyrus (all *p* < 0.02). Considering the high collinearity between some of these respective BP_ND_ values, we included for the present study the individual ^18^F-AV1451 binding of thalamus, putamen and cerebellar dentate gyrus for correlational analyses with multimodal MRI imaging. However, as tau deposition is prominent in other PSP-specific regions, we performed the same correlational analyses using ^18^F-AV1451 binding in the midbrain, frontal lobe, and the average of all cortical ROIs.

### Statistical analyses

Demographic data were analyzed with Stata software Version 14.2 (College Station, TX). Assessment of distribution for continuous variables was performed with Shapiro–Wilk test and visualization of histogram plots, followed by t test or Mann–Whitney *U*, accordingly. Categorical variables were compared with Chi-Square test. Statistical significance was considered when *p* < 0.05.

GM volume and CTh comparisons between PSP subjects and controls were performed in CAT12 with a 2-sample *t* test using age and gender as covariates. For VBM, total intracranial volume (TIV) was also added as a covariate. Correlation of voxelwise GM volume/CTh maps with regional ^18^F-AV1451 BP_ND_ was performed using multiple regressions with individual ^18^F-AV1451 binding in the thalamus, putamen, and dentate gyrus as the variable of interest, and age, gender, PSP-RS, and TIV (only for VBM) as covariates. A significant statistical threshold of *p* < 0.05 false discovery rate (FDR)-corrected was considered. As the present study involves relatively modest groups of subjects, we also showed uncorrected *p* < 0.001 results using an extent threshold (k) of the expected voxels/vertices per cluster.

Regarding DTI, the randomise function in FSL was implemented to identify group differences in DTI metrics between PSP and controls, using an independent sample *t* test, with age and gender as covariates. Similarly, voxelwise correlational models between DTI measures and average ^18^F-AV1451 binding in specific ROIs for PSP subjects included age, gender, PSP-RS as covariates. Statistical significance was determined using non-parametric permutation testing (*n* = 10′000 permutations), applying threshold-free cluster enhancement (TFCE) and adjusting for multiple comparisons with familywise error (FWE) *p* < 0.05. Anatomical labelling of significant TBSS white matter clusters was facilitated using the John Hopkins—ICBM white matter atlas, available as part of the FSL package.

For PSP subjects, correlation analyses between clinical scales (ACER and PSP-RS) and regional ^18^F-AV1451 BP_ND_ or GM/CTh/DTI maps were performed using multiple linear regressions using the same covariates as above (age and gender + TIV for VBM). We also performed Spearman correlation between clinical scores and regional ^18^F-AV1451 binding.

## Results

### Demographics

Demographic and clinical characteristics of PSP and controls are shown in Table 1. Both groups were comparable in terms of age, gender, and education attainment. As expected, cognitive scores (i.e., MMSE and ACER) were lower in the PSP group (*p* = 0.01 and *p* < 0.001, respectively). Average disease duration at scan for the PSP group was 4.6 ± 2.1 years (range 1.5–9).

**Table 1:** Clinical characteristics of included subjects

	PSP (*n* = 23)	Controls (*n* = 23)	*P* value
Age, years	68.8 ± 5.8 (52–79)	68.7 ± 7.3 (55–81)	0.95*
Female participants	39% (9/23)	48% (11/23)	0.55^§^
Education, years	12.2 ± 2.1 (10–17)	13.5 ± 2.5 (9–17)	0.06*
MMSE	26.0 ± 4.5 (13–30)	28.7 ± 1.1 (26–30)	0.01^#^
ACER	78.2 ± 16.5 (36–95)	91.3 ± 6.3 (75–99)	< 0.001^#^
PSP-RS	48.0 ± 16.3 (25–77)	–	NA
Disease duration at scan, years	4.6 ± 2.1 (1.5–9)	–	NA

Values are mean ± standard deviation (range)

*Student *t* test

^§^Chi-square test

^#^Mann–Whitney *U* test

*NA* not applicable

### GM/CTh/DTI group comparisons

**Fig. 1:**
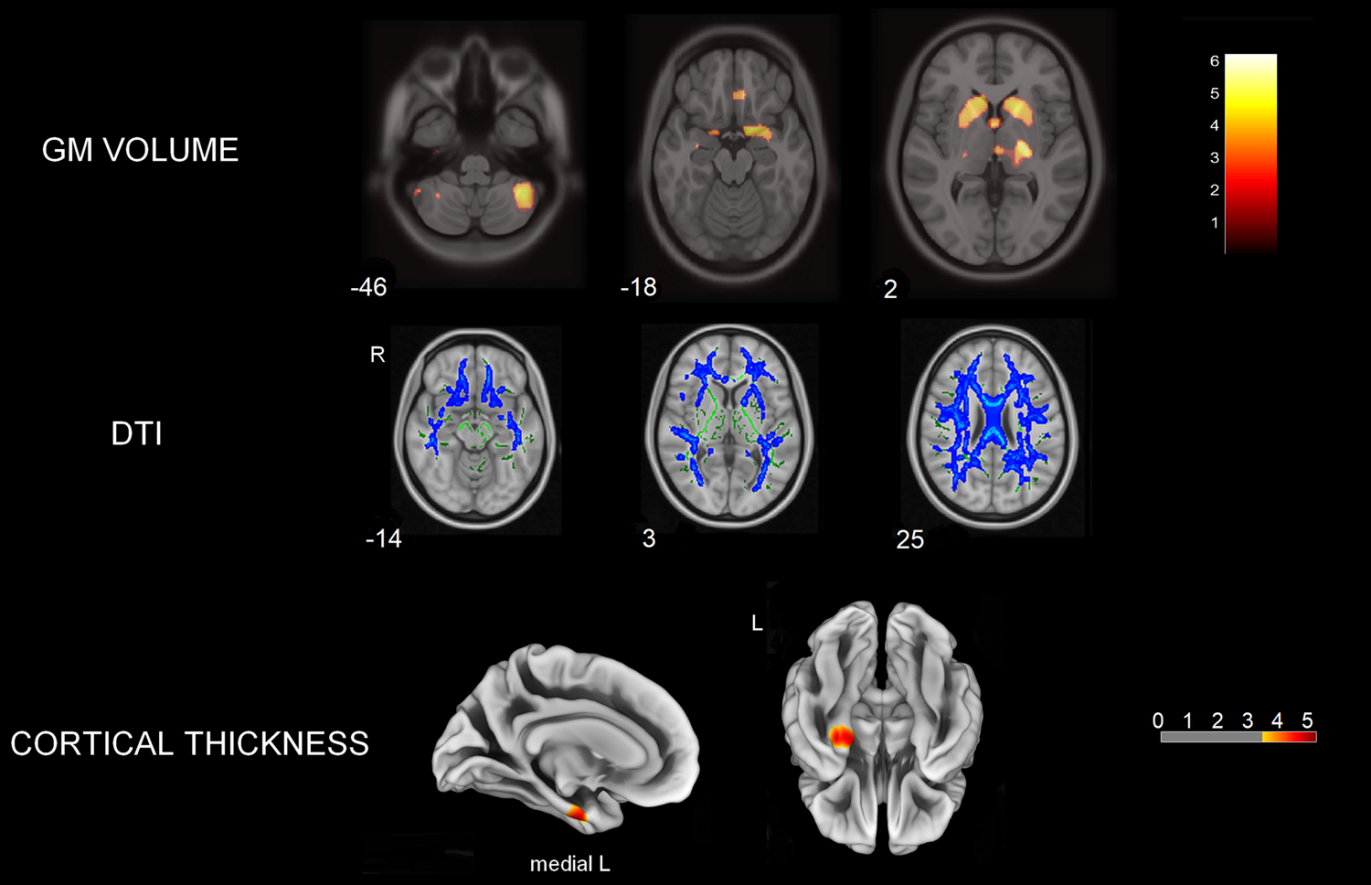
Multimodal MRI/DTI group comparisons between PSP and controls showing GM volume loss (red), DTI changes (increased RD, blue), and cortical thinning (yellow–red) for PSP subjects. Color bars represent T-scores

Comparisons between groups revealed GM volume loss for PSP subjects in both cortical and subcortical regions, including right medial frontal, right temporal pole, left supplementary motor area (SMA), as well as bilateral caudate nucleus, putamen, thalamus, midbrain, and cerebellum (FDR-corrected *p* < 0.05). In addition, we observed reduced CTh for PSP subjects in the left entorhinal and fusiform gyri (uncorrected *p* < 0.001, *k* = 41 vertices). DTI analyses confirmed that relative to controls, PSP subjects had diffuse white matter damage in the corpus callosum (genu, body, and splenium), bilateral internal capsule, corona radiata, posterior thalamic radiations, cingulate white matter, superior longitudinal fasciculus (SLF), sagittal stratum, and uncinate fasciculus (FWE-corrected *p* < 0.05). These abnormalities were consistent across the three DTI metrics (i.e., decreased FA, increased MD and RD for PSP) (Fig. 1).

### Voxelwise correlation of structural MRI/DTI imaging and ^18^F-AV1451 binding

**Fig. 2:**
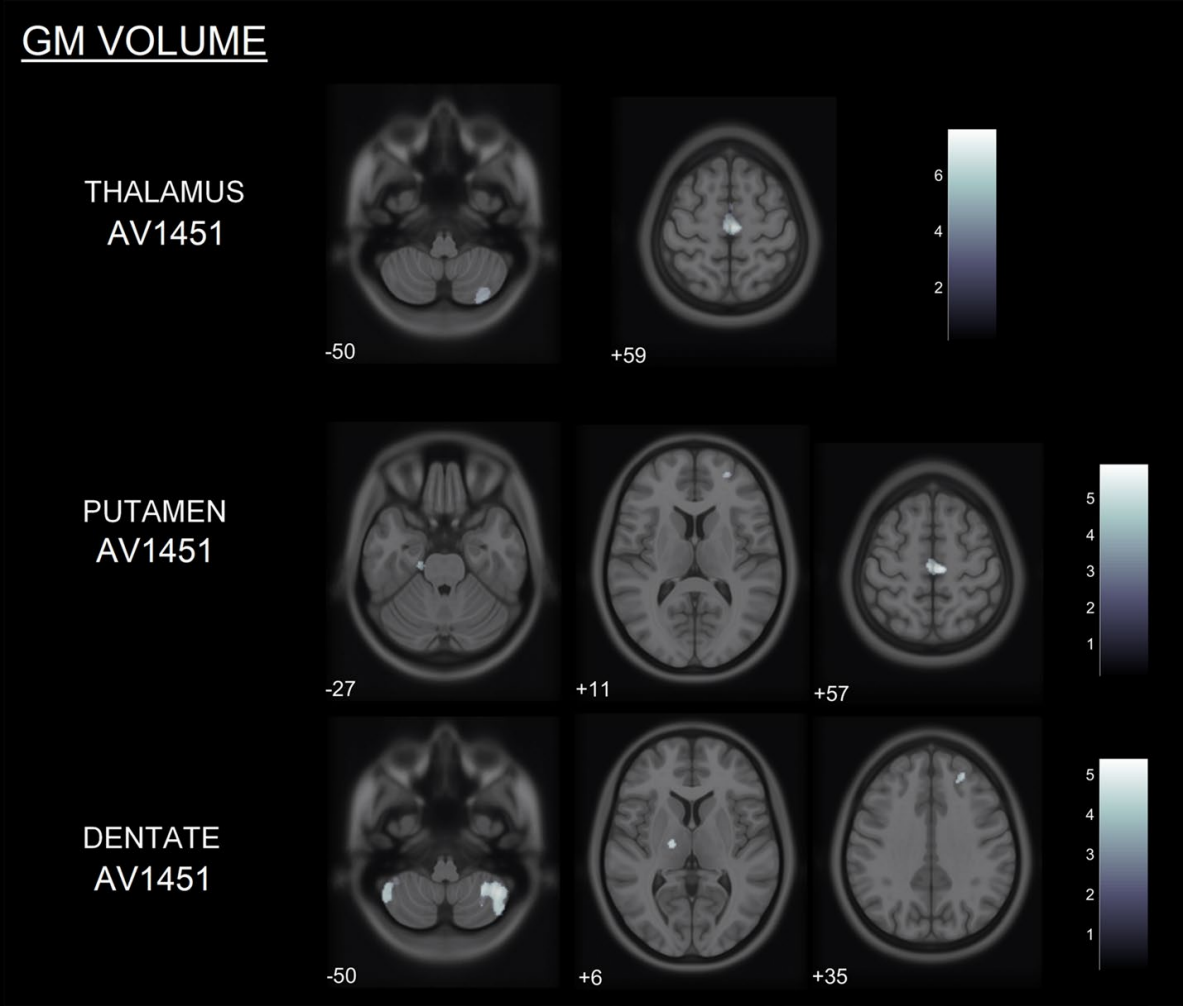
GM volume loss (grey) in relation to thalamic, putaminal and dentate ^18^F-AV1451 BP_ND_ in PSP subjects (*p* < 0.001, *k* = 20 voxels). Color bars are T-scores

**Fig. 3:**
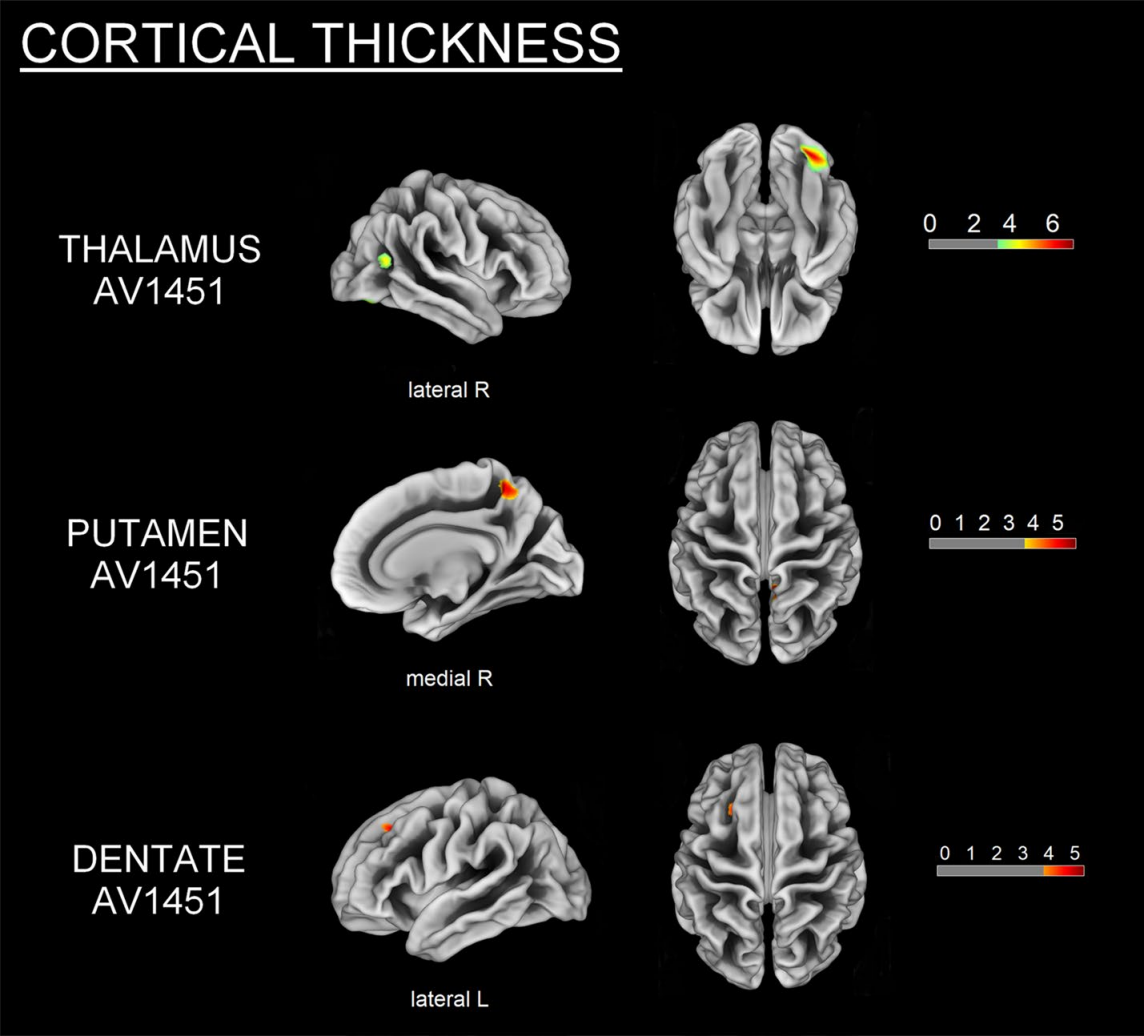
CTh reduction (yellow–red) related to thalamic, putaminal and dentate ^18^F-AV1451 BP_ND_ in PSP subjects (*p* < 0.001, *k* = 36 vertices). Color bars are T-scores

**Fig. 4:**
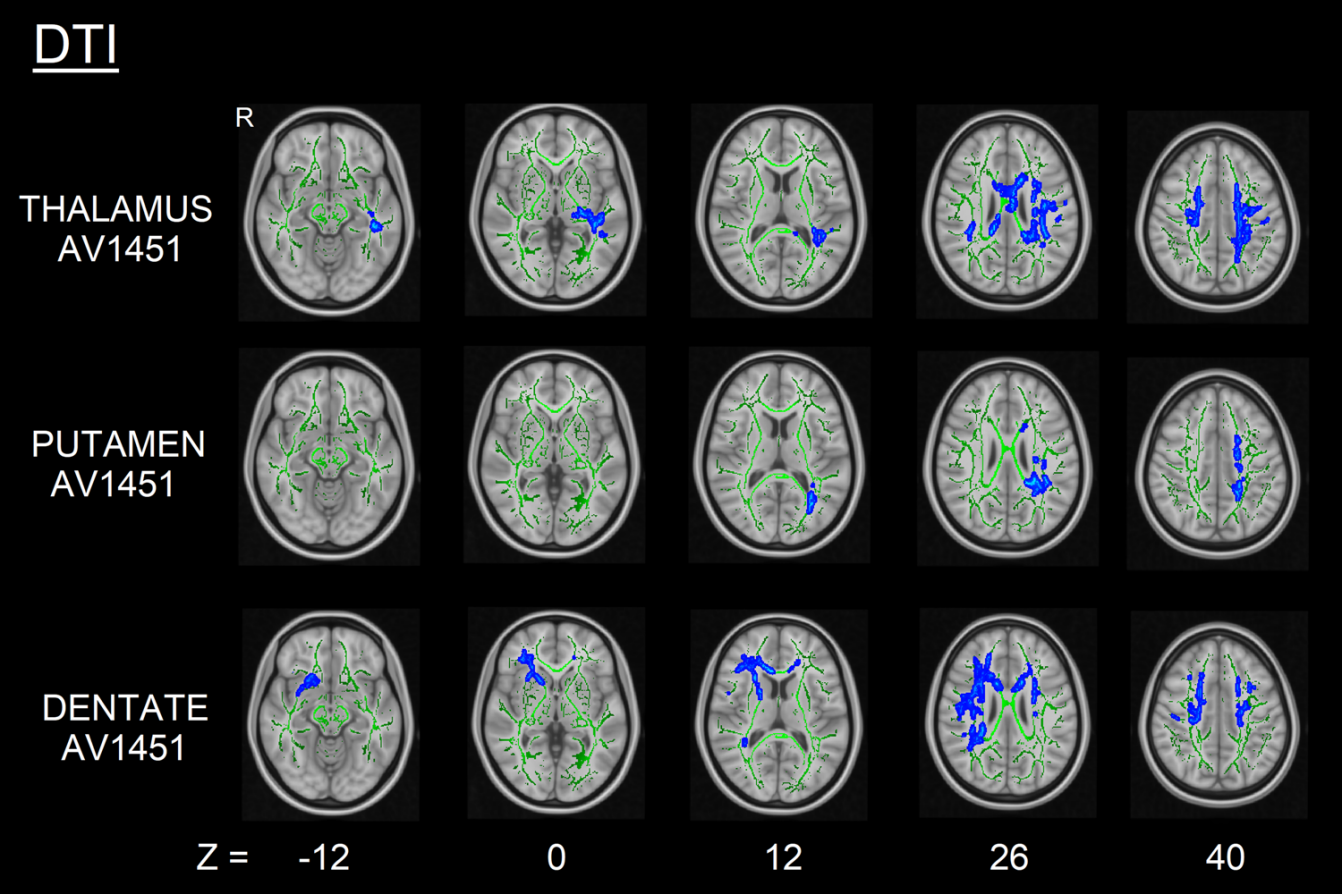
DTI changes (increased RD, blue) correlated with thalamic, putaminal, and dentate ^18^F-AV1451 BP_ND_ in PSP subjects (FWE-corrected *p* < 0.05)

Higher thalamic, putaminal, and cerebellar dentate ^18^F-AV1451 BP_ND_ were correlated with lower GM volume (all uncorrected *p* < 0.001, *k* = 20 voxels) (Fig. 2), more pronounced cortical thinning (all uncorrected *p* < 0.001, *k* = 36 vertices) (Fig. 3) and altered white matter metrics (e.g., increased MD/RD or decreased FA) (FWE-corrected *p* < 0.05) (Fig. 4). More specifically, higher thalamic ^18^F-AV1451 BP_ND_ was associated with GM volume reduction in bilateral SMA, precentral gyrus, and right cerebellum; decreased CTh in right latero-occipital, fusiform, and inferior parietal gyri; and DTI alterations in bilateral internal capsule, corona radiata, posterior thalamic radiations, SLF, body of corpus callosum, cingulate white matter, and superior fronto-occipital fasciculus.

Higher putaminal ^18^F-AV1451 BP_ND_ was correlated with lower GM volume in bilateral precentral gyrus, left SMA and parahippocampal gyrus, as well as right superior and middle frontal gyri; reduced CTh in the right precuneus and DTI alterations in bilateral internal capsule, corona radiata, cingulate white matter, and SLF.

Finally, higher ^18^F-AV1451 BP_ND_ in the cerebellar dentate gyrus was correlated with GM volume loss in bilateral cerebellum and precentral gyrus, right superior and middle frontal gyri, and left thalamus; CTh reduction in left superior and middle frontal gyri; and DTI alterations in genu and body of corpus callosum, bilateral corona radiata, SLF and superior fronto-occipital fasciculus, right internal capsule, posterior thalamic radiations, and cingulate white matter.

Correlational analyses were additionally performed using ^18^F-AV1451 binding in the midbrain, frontal lobe, and global cortical level to assess whether these PSP-prone regions in terms of tau pathology had specific structural impairment related to their ^18^F-AV1451 binding. Higher midbrain ^18^F-AV1451 was related to right temporal GM reduction, whereas frontal and global binding was negatively correlated with bilateral superior frontal and precentral GM reduction (*p* < 0.001, *k* = 20 voxels). No significant correlation was observed between these three ROI’s ^18^F-AV1451 and cortical thinning (*p* > 0.001), whereas their ^18^F-AV1451 binding was associated with altered DTI (increased RD) in body of corpus callosum and motor tracts (FWE *p* < 0.05).

### Clinical correlations with ^18^F-AV1451 and MRI imaging

Higher PSP-RS score correlated with GM volume loss in bilateral middle cingulate gyrus, right middle temporal gyrus, left fusiform and middle occipital gyri, bilateral cuneus, and left cerebellum (*p* < 0.001, *k* = 21 voxels). A higher PSP-RS score was also associated with increased RD in white matter facing bilateral middle temporal gyrus, right lateral occipital gyrus and cuneus (*p* < 0.001, *k* = 25 voxels). There was no significant correlation with CTh and ^18^F-AV1451 BP_ND_. Using Spearman correlation of ^18^F-AV1451 and PSP-RS, we observed a trend between higher PSP-RS and higher midbrain ^18^F-AV1451 binding (rho = 0.34, *p* = 0.13).

Lower ACER score was correlated with lower GM volume in bilateral medial temporal regions (hippocampus, amygdala, and entorhinal cortex), bilateral temporal poles, right superior frontal gyrus, thalamus, and pallidum (*p* < 0.001, *k* = 20 voxels). A lower ACER was also related to CTh reduction in left superior and middle temporal gyrus (*p* < 0.001, *k* = 37 vertices), as well as white matter deficits (increased RD) in the genu and body of corpus callosum, bilateral internal capsule and corona radiata, right cingulate white matter and SLF (FWE-corrected *p* < 0.05). We found a trend between higher ACER score and lower ^18^F-AV1451 binding in dentate gyrus using Spearman correlation (rho = − 0.32, *p* = 0.16).

## Discussion

Using multimodal MRI imaging with VBM, SBM and DTI, we demonstrate that higher ^18^F-AV1451 binding in tau-prone regions affected by PSP was correlated with changes in grey and white matter. We interpret these findings in terms of a correlation between tau pathology and structural integrity while recognizing important caveats below.

In comparing PSP patients with controls independently of ^18^F-AV1451 binding, extensive structural impairment was observed, with GM volume loss affecting frontotemporal regions, basal ganglia, midbrain and cerebellum, CTh reduction in left entorhinal and fusiform gyri, and DTI changes in the corpus callosum, internal capsule, corona radiata, and posterior thalamic radiations, among others. These results are in keeping with the previous observations [12–14, 17, 18], suggesting a key role of grey and white matter changes in the pathophysiology of PSP, especially the PSP-Richardson syndrome [26].

We also observed that ^18^F-AV1451 in high-binding regions for PSP (i.e., thalamus, putamen, and cerebellar dentate gyrus) was correlated with GM volume loss mainly in anterior regions, i.e., SMA, superior and middle frontal and precentral gyri, while increased PET binding was related to cortical thinning in posterior regions (e.g., lateral-occipital, fusiform gyrus and precuneus). In addition, ^18^F-AV1451 BP_ND_ was associated with altered DTI in motor tracts (corona radiata, internal capsule), posterior thalamic radiations and SLF, the latter being particularly involved in spatial attention, oculomotor function, and motor behaviour [27]. Higher ^18^F-AV1451 binding in the thalamus and dentate gyrus was also related to white matter disruption in the body of corpus callosum, whose fibres pass through the corona radiata to reach the brain surface, the cingulum (which is densely connected to the thalamus and spinothalamic tract) and superior fronto-occipital fasciculus. These cross-modal association shed light onto the intricate in vivo relationship between the integrity of connecting fibres and ^18^F-AV1451 binding. Due to the cross-sectional design of our study, it is still unclear whether microstructural damage is a direct consequence of tau pathology in the white matter (as observed post mortem) or consecutive to Wallerian degeneration [28]. However, considering the GM volume loss and DTI signal impairment taking place in similar anterior regions in relation to higher ^18^F-AV1451 binding, our results suggest that white matter degeneration is at least partly secondary to GM volume loss. Using a sparse canonical correlation analysis between DTI and ^18^F-AV1451 PET imaging, Sintini et al. recently demonstrated that higher ^18^F-AV1451 binding in cerebellar dentate, red nucleus and subthalamic nucleus was associated with altered DTI in sagittal stratum, corona radiata and superior cerebellar peduncle [29].

At variance with GM volume and DTI changes observed in anterior brain regions, increased ^18^F-AV1451 binding was associated with cortical thinning in more posterior (parieto-occipital) areas. These findings are of particular interest as CTh reduction in PSP is usually described in frontal regions, with little changes observed in longitudinal studies [16]. Therefore, with the present correlational analyses, we were able to tackle more subtle posterior cortical changes in relationship to a PET proxy assessing tau deposition.

In addition to these three regions (i.e., putamen, thalamus, and dentate gyrus), we performed correlational analyses of MRI changes with midbrain, frontal lobe, and global cortical ^18^F-AV1451 binding, as they are PSP-prone regions for tau deposition. Although unspecific white matter changes involving corpus callosum and motor tracts were related to higher ^18^F-AV1451 uptake in these ROIs, there was no significant cortical thinning in relationship to it. In addition, higher frontal and global ^18^F-AV1451 was negatively correlated with frontal GM volume loss. This highlights the fact that the three high-binding ^18^F-AV1451 regions we initially selected (i.e. thalamus, putamen, and dentate gyrus) have specific relationships with structural GM loss, as remote effects on GM involving both cortical thinning and volume loss are specifically related to higher ^18^F-AV1451 binding in diencephalic and cerebellar regions. One must insist that these are spatiotemporal relationships using a cross-sectional approach and that it does not necessarily involves causality. However, considering our modest cohort and a relatively stringent significance threshold, we believe that this brings novel insights into the complex relationship of a tau PET proxy with structural changes.

With regard to multimodal imaging correlates of clinical motor (PSP-RS) or cognitive (ACER) impairment, our results pointed out to a lack of correlation with ^18^F-AV1451 binding. This is in accordance with Schonhaut et al. [7] but contrasts with Smith et al. [9], who found a correlation between pallidal ^18^F-AV1451 binding and PSP-RS using Spearman correlations. One explanation could be the relatively small sample sizes or early disease stage of our patients. In addition, we assessed correlation using linear regressions adjusting for age and gender. In fact, when performing a Spearman correlation instead, we observed a trend between higher midbrain AV1451 and higher PSPRS score (rho = 0.34, *p* = 0.13). In addition, lower ^18^F-AV1451 binding in the dentate gyrus was associated at the trend level with higher ACER (rho = − 0.32, *p* = 0.16). In AD, ^18^F-AV1451 binding in the temporal cortex was negatively related to temporal (especially its medial portion) CTh change [30]. In addition, Bejanin et al*.* [31] showed that tau deposition was related in a region-specific manner to cognitive decline. That ^18^F-AV1451 is a more sensitive marker of paired helical filaments than straight filamentous might also play a role.

Conversely, we did find that lower cognitive performance according to ACER score was significantly associated with reduced GM volume in mediotemporal regions, right superior frontal gyrus, thalamus, and pallidum. In addition, lower ACER correlated with impaired diffusion metrics in the corpus callosum, cingulum, motor tracts, and SLF. These highlight the prominent role of frontal dysfunction in PSP [32, 33], as these white matter tracts are densely connected to the frontal lobe [34]. In a previous study, Piattella et al*.* showed that MMSE score in PSP was correlated with whole white matter mean FA [18].

While PSP-RS score was not associated with specific cortical thinning, we observed altered GM and DTI in bilateral temporal and right occipital regions, highlighting the close relationship between atrophy and white matter degeneration according to motor impairment.

Our study is not without limitations. First, our results would require confirmation in larger and independent samples. Second, the present relationship between ^18^F-AV1451 and structural imaging is based on a cross-sectional design: longitudinal studies are, therefore, required to fully assess the spatial and temporal interplay between grey/white matter integrity and ^18^F-AV1451 in PSP, and test causal models of pathology in humans. Moreover, due to the relatively modest sample size, most of our correlational results were uncorrected for multiple comparisons. We, however, used a stringent significance threshold (*p* < 0.001) and extent cluster threshold. In addition, ^18^F-AV1451 off-target binding is a caveat to our interpretation. Neuromelanin-containing cells in the substantia nigra and monoamine oxidase in the striatum are bound by this ligand [35, 36]. However, we recently showed that off-target binding in basal ganglia, cortex or adjacent white matter is not sufficient an explanation, as postmortem data did not show relevant neuromelanin-containing cells [6]. The very high clinic–pathological correlations in PSP–Richardson syndrome preclude TDP43 [11] or concurrent AD pathology as an alternative explanation, noting that the six patients in our study who have come to post-mortem examination were confirmed as PSP without significant dual pathology.

In conclusion, we present evidence of the in vivo association between structural brain integrity and ^18^F-AV1451 in PSP. Longitudinal studies would be helpful to determine the dynamic changes occurring for both tau deposition and grey/white matter changes. Tau PET probes more specific and sensitive for straight filaments would also enable confirmation of the complex interplay between cortical tau aggregation, grey/white matter degeneration and disease progression in PSP.
